# Altered sodium channel-protein associations in critical illness myopathy

**DOI:** 10.1186/2044-5040-2-17

**Published:** 2012-08-30

**Authors:** Susan D Kraner, Kevin R Novak, Qingbo Wang, Junmin Peng, Mark M Rich

**Affiliations:** 1 Department of Neuroscience, Cell Biology, and Physiology, Wright State University School of Medicine, 3640 Colonel Glenn Hwy, Dayton, OH, 45435, USA; 2 Department of Structural Biology, St. Jude’s Children Research Hospital, Memphis, TN, 38105, USA

**Keywords:** Skeletal muscle, Na_V_ 1.4 sodium channel, Na_V_ 1.5 sodium channel, Nitric oxide (NO), Neuronal nitric oxide synthase (nNOS), Glycosylation, Phosphorylation, Action potential, Excitability, Denervation

## Abstract

**Background:**

During the acute phase of critical illness myopathy (CIM) there is inexcitability of skeletal muscle. In a rat model of CIM, muscle inexcitability is due to inactivation of sodium channels. A major contributor to this sodium channel inactivation is a hyperpolarized shift in the voltage dependence of sodium channel inactivation. The goal of the current study was to find a biochemical correlate of the hyperpolarized shift in sodium channel inactivation.

**Methods:**

The rat model of CIM was generated by cutting the sciatic nerve and subsequent injections of dexamethasone for 7 days. Skeletal muscle membranes were prepared from gastrocnemius muscles, and purification and biochemical analyses carried out. Immunoprecipitations were performed with a pan-sodium channel antibody, and the resulting complexes probed in Western blots with various antibodies.

**Results:**

We carried out analyses of sodium channel glycosylation, phosphorylation, and association with other proteins. Although there was some loss of channel glycosylation in the disease, as assessed by size analysis of glycosylated and de-glycosylated protein in control and CIM samples, previous work by other investigators suggest that such loss would most likely shift channel inactivation gating in a depolarizing direction; thus such loss was viewed as compensatory rather than causative of the disease. A phosphorylation site at serine 487 was identified on the Na_V_ 1.4 sodium channel α subunit, but there was no clear evidence of altered phosphorylation in the disease. Co-immunoprecipitation experiments carried out with a pan-sodium channel antibody confirmed that the sodium channel was associated with proteins of the dystrophin associated protein complex (DAPC). This complex differed between control and CIM samples. Syntrophin, dystrophin, and plectin associated strongly with sodium channels in both control and disease conditions, while β-dystroglycan and neuronal nitric oxide synthase (nNOS) associated strongly with the sodium channel only in CIM. Recording of action potentials revealed that denervated muscle in mice lacking nNOS was more excitable than control denervated muscle.

**Conclusion:**

Taken together, these data suggest that the conformation/protein association of the sodium channel complex differs in control and critical illness myopathy muscle membranes; and suggest that nitric oxide signaling plays a role in development of muscle inexcitability.

## Background

Critical illness myopathy (CIM) is the most common cause of severe weakness in patients in the intensive care unit [1,2]. One of the hallmarks of the acute phase of CIM is paralysis due to loss of muscle’s electrical excitability [3,4]. To determine the mechanism underlying loss of excitability, we utilize a rat model of CIM in which corticosteroid treatment is combined with denervation to mimic treatment of critically-ill patients with corticosteroids and neuromuscular blocking agents [5,6]. In the rat model, inactivation of sodium channels is the cause of inexcitability [7,8]. A major contributor to the increase in inactivation is a hyperpolarized shift in the voltage dependence of inactivation of the adult (Na_V_ 1.4) and embryonic (Na_V_ 1.5) skeletal muscle sodium channel isoforms expressed in CIM muscle [9]. As the rat model of CIM occurs in genetically normal rats, this hyperpolarized shift in sodium channel gating must be due to post-translational modification of sodium channels or an alteration in sodium channel association with other proteins that modify gating.

The goal of this study was to identify biochemical changes that could potentially underlie the hyperpolarized shift in the voltage dependence of sodium channel inactivation in CIM. We found changes in several candidates, although at least one change appeared compensatory rather than causative. We identified an increase in the amount of neuronal nitric oxide synthase (nNOS) associated with the sodium channel in CIM as a change that might underlie the hyperpolarized shift in the voltage dependence of inactivation.

## Methods

### Preparation of control and critical illness myopathy muscle samples

All animal protocols were approved by the Institutional Animal Care and Use Committee at Wright State University. Critical illness myopathy was induced by a combination of denervation and dexamethasone treatment. Briefly, rat muscle was denervated by removing a 10-mm segment of the sciatic nerve in isoflurane-anesthetized adult female Wistar rats (250 to 350 g body weight). Buprenorphine was administered subcutaneously for postoperative analgesia. Dexamethasone (4 mg kg ^-1^) and antibiotics (0.2 mL of 2.27% Baytril, Bayer, Shawnee Mission, KS, USA) were administered intraperitoneally beginning on the day of denervation and continuing for 7 days. Rats were killed by carbon dioxide inhalation, and the tibialis anterior, soleus, and gastrocnemius muscles removed, weighed, and snap-frozen in liquid nitrogen. For control muscles, non-treated rats from the same group were used and muscles harvested in the same way. All muscles were stored at −80°C until use.

### Preparation of muscle membranes and classical purification of sodium channels

Membranes were prepared from the gastrocnemius muscles as previously reported [10]. Samples were thawed on ice for 10 min and minced finely on a glass plate on ice. Samples were transferred to tubes containing sucrose buffer with protease and phosphatase inhibitors, using 10 volumes of buffer per gram of tissue. The sucrose buffer contained 0.3 M sucrose, 75 mM NaCl, 10 mM EGTA, 10 mM EDTA, 10 mM Tris, pH 7.4, 1 mM phenylmethylsulfonyl fluoride (PMSF), 1 mM iodoacetamide, 0.1 μg/mL pepstatin A, 10 μM leupeptin, 2.5 μM ALLN, and 1 mM sodium fluoride. Samples were homogenized using a PowerGen 700 tissue homogenizer at a setting of 6 for 30 s. The samples were centrifuged in a SS34 rotor at 4,500 g for 15 min. The supernatants were removed and centrifuged again. The final supernatants were centrifuged in a SW55 rotor at 138,000 g for 1 h. The pellets from this last step were resuspended in fresh sucrose buffers with inhibitors and used as the membrane fraction.

For classical purification of sodium channels, membranes were solubilized using NP40 and the sodium channel fraction sequentially purified on DEAE-Sepharose and wheat germ agglutinin-Sepharose columns as described [11]. For all membrane preparation and channel purification procedures, samples, equipment, and buffers were kept at 0-4°C.

### SDS-PAGE, western blot analysis, and antibodies

Membrane fractions were assayed for protein content using the Lowry protein assay, and equal amounts of protein were loaded for control and CIM samples for each protein assessed (approximately 40 μg protein per lane). For deglycosylation experiments and co-immunoprecipitation experiments, the same amount of membrane protein was used to initiate the experiment; and equal volumes of the final product were loaded for each control and CIM sample analyzed. SDS-PAGE was performed on Criterion 4% to 20% acrylamide gels for most samples; but 5% gels were used for the deglycosylation experiments, and 10% to 14.5% gels were used for analysis of syntrophin and β-dystroglycan co-immunoprecipitations to better resolve the molecular weight region being analyzed. Western blot transfers were carried out in a Criterion transfer apparatus using nitrocellulose as the blotting medium. Following transfer, blots were washed with PBS/0.1% Tween, blocked in 1% I-Block (Tropix) in PBS/0.1% Tween for 1 h; incubated with first antibody for 2 h; washed and incubated with mouse or rabbit secondary antibody conjugated to alkaline phosphatase (Tropix); and visualized using CDP-Star (Tropix) and a Fujifilm LAS-3000 close-caption device camera.

A pan-sodium channel antibody (pan Na_V_ 1.x) was raised against the highly conserved peptide, TEEQKKYYNAMKKLGSKK, corresponding to SP19 [12] at Open Biosystems, ThermoFisher Scientific. The affinity-purified antibody selectively reacted to sodium channel in skeletal muscle and other tissues and could be displaced by the peptide antigen in western blot analysis. The antibody to FGF13 was provided by Dr Geoffrey Pitts (Duke University). The Na_V_ 1.4-specific monoclonal antibody was provided by Dr Robert L Barchi (Thomas Jefferson Medical School), and can be obtained commercially from Sigma (S9568). Other antibodies were obtained from the following commercial sources: FGF12 (Abgent AP6750b), plectin (Epitomics 1399–1), dystrophin (Vector Labs VP-D508), Na_V_ 1.5 (Alomone ASC-005), nNOS (Invitrogen 37–2800), β-dystroglycan (Abcam ab 49515), and syntrophin (Abcam ab11425).

Some gels were stained rather than processed for western blots. For analysis of phosphoproteins, Peppermint Stick Phosphoprotein Molecular Weight Standards (Invitrogen), which include both phosphoprotein and non-phosphoprotein standards, were included on the gels as controls. After electrophoresis, gels were fixed in 50% methanol and 10% acetic acid for 30 min, transferred to the Pro-Q Diamond Phosphoprotein Stain (Invitrogen) for 90 min, then transferred to Destain (Invitrogen) for 30 min. The gel was de-stained twice more for 30 min; then washed twice in ultrapure water. The gel was imaged on a flatbed fluorescent scanner (Fujifilm FLA-5100). After the phosphoprotein stain, the gel was immediately processed for all-protein stain with SYPRO Ruby Protein Stain (Invitrogen) overnight. The gel was de-stained with 10% MeOH and 7% acetic acid for 45 min, washed twice in ultrapure water, and imaged on the fluorescent scanner. The containers with the gels were covered with foil to prevent photo-bleaching of the stains.

For proteins that were purified using gels, the gels were not fixed prior to the SYPRO stain. After the water wash, these gels were imaged on a UV light-box, their pictures taken with a digital camera, and the bands of interest excised and sent to the proteomics facility.

### Deglycosylation

For deglycosylation with PNGase F (New England Biolabs), 100 μg of control or CIM membranes was adjusted to 80 uL volume; then 20 uL of 5X denaturation mix (5 mM PMSF, iodoacetamide, NaF, 12.5 μM ALLN, 0.5 μg/mL pepstatin A, 2.5% SDS, and 0.2 M DTT) was added and the sample incubated at 65°C for 20 min. Following denaturation and cooling to room temperature, 20 uL of 10% NP40, 20 μL of 500 mM sodium phosphate (pH 7.5), 59 μL water, and 1 μL (500 units) of PNGase F were added to each sample and the samples incubated at 37°C for 1 h. Samples were quenched by addition of gel sample buffer. For neuraminidase (New England Biolabs) treatments, 10 μg of control or CIM membranes were adjusted to 100 μL with 10 μL of 500 mM sodium citrate (pH 6.0), 10 μL of 10% NP40, 5 μL neuraminidase (250 units), and water. Samples were heated at 37°C for 4 h. As a control, 10 μg of fetuin was treated in the same way. Samples were quenched by addition of gel sample buffer. For all samples, mock treatments without enzyme were also carried out. Samples were analyzed by western blot with Na_V_ 1.4-specific antibody.

### Analysis of phosphorylation by tandem mass spec

The mass spectrometric analysis was performed on an optimized proteomics platform as previously reported [13]. Briefly, protein gel bands were washed and subjected to standard in-gel trypsin digestion. Digested peptides were analyzed by capillary reverse-phase liquid chromatography coupled with tandem mass spectrometry (LC-MS/MS), in which the eluted peptides were analyzed by an MS survey scan followed by 10 data-dependent MS/MS scans on an LTQ-Orbitrap ion trap mass spectrometer (Thermo Scientific). Acquired MS/MS spectra were then searched against the rat reference database of the National Center for Biotechnology Information with differential modifications on serine/threonine/tyrosine (+79.9663 Da); then filtered by matching scores and mass accuracy to reduce protein false discovery rate to less than 1% using a concatenated reversed database [14]. Furthermore, we manually validated MS/MS spectra of matched phosphopeptides using the following criteria: (1) the presence of signature phosphate neutral losses (−49 for doubly charged or −32.7 for triply charged) for serine/threonine phosphorylation; (2) the strong intensity (usually the top peak) of the neutral loss peak; (3) possible ambiguity of modification site assignment; and (4) comparison of the MS/MS spectra of modified peptides with the corresponding unmodified counterparts.

### Immunoprecipitation

For each sample analyzed (control, CIM, control/peptide, CIM/peptide), 200 μL of protein G dynabeads (Invitrogen) was incubated with 40 μg of pan-Na_V_ 1.x antibody - and for the peptide controls, 40 μg of blocking peptide - for 3 h to 4 h, rotating end-over-end. During this incubation, 3.0 mg of membrane protein was prepared for each sample. In a total of 1.8 mL volume, the sample was solubilized in a buffer containing 20 mM KPO4 (pH 6.5), 180 mM KCl, 1 mM EGTA, 0.5 mM MgCl2, 1 mM PMSF, 1 mM iodoacetamide, 1 mM NaF, 2.5 μM ALLN, 10 μM leupeptin, 0.1 μg/ml pepstatin A, and 1% NP40. Samples were vortexed, incubated on ice for 1 h, centrifuged at 13,700 x g in a refrigerated microfuge for 30 min, and the supernatants recovered for the immunoprecipitation. The antibody-bound beads were washed twice with PBS/0.5% NP40 and incubated with the solubilized channel protein, rotating end-over-end, overnight. The next morning, the beads were collected at the side of the tube using a magnet, and the supernatants removed. The beads were washed five times with the same buffer used for solubilization, except NP40/asolectin was used in the buffer instead of NP40. The samples were eluted from the beads with gel sample buffer. Equal volumes of sample were loaded for each sample (con IP, CIM IP, con IP/pep, CIM IP/pep), and starting membranes were used as a positive control (con memb, CIM memb). Six separate immunoprecipitations were carried out. Prior to eluting with sample buffer, all procedures were maintained at 0°C to 4°C.

### Immunohistochemistry

Medial gastrocnemius muscles from control or CIM rats were removed, fixed in 4% paraformaldehyde for 1 h, cryoprotected in 15% sucrose solution overnight, and frozen in liquid nitrogen. Ten-μm-thick cross-sections were cut. The same rabbit FGF12 and mouse monoclonal nNOS antibodies used in western analysis were used for staining. For dystrophin staining of the FGF12 samples, the same mouse monoclonal used in western analysis was used, but for analysis of the mouse nNOS-stained samples, a rabbit dystrophin was used. Labeling of mouse monoclonal antibodies was visualized using a Dylight 488-conjugated donkey anti-mouse secondary antibody (Jackson ImmunoResearch Laboratories). Labeling of rabbit antibodies was visualized using a rhodamine-conjugated donkey anti-rabbit antibody (Jackson ImmunoResearch Laboratories). Images were obtained using a Fluoview FV 1000 confocal microscope and an X60 oil objective (Olympus Optical).

### Electrophysiology

Wild type and nNOS knockout adult mice (B6.129 S4-*Nos1*^*tm1Plh*^ /J, Jackson Labs, 25 g to 30 g body weight) were denervated by removing a 0.5-mm segment of the left sciatic nerve in the upper thigh under isoflurane anesthesia (2% to 3% inhaled). Buprenorphine was administered subcutaneously for postoperative analgesia. Mice were sacrificed on days 3 or 7 by carbon dioxide inhalation. The extensor digitorum longus (EDL) muscle was dissected tendon to tendon; then muscle fibers were labeled with 10 μM 4-Di-2-ASP, and imaged using an upright epifluorescence microscope during recording of action potentials as previously described [8]. For all experiments, the recording chamber was continuously perfused with solution containing (in millimoles per liter) NaCl, 118; KCl, 3.5; CaCl_2_, 1.5; MgSO_4_, 0.7; NaHCO_3_, 26.2; NaH_2_PO_4_, 1.7; and glucose, 5.5 (pH 7.3-7.4, 20-22°C) equilibrated with 95% O_2_ and 5% CO_2_.

### Statistical analysis

Western blots were quantified using the software supplied with the Fujifilm LAS-3000 close-caption device camera. For western blots with multiple samples of control and CIM samples (for Na_V_ 1.4, Na_V_ 1.5, pan-Na_V_ 1.x, FGF12, and FGF13), the average of the control was used as the 100% standard. All individual control and CIM samples were calculated relative to this number, and errors shown are SEM. Statistical comparison between control and CIM were carried out using Student’s *t*-test. For quantification of the co-immunoprecipitation, the CIM was calculated relative to the control (set at 100%) for each individual protein in each co-immunoprecipitation. The expression in CIM relative to control for all six co-immunoprecipitations was analyzed by Student’s *t*-test, and errors shown are SEM. Muscle excitability was compared by calculating the percent of inexcitable fibers in each muscle. At least six fibers were studied in each muscle. The percent for each muscle was compared between control and nNOS-null mice using Student’s *t*-test with n as the number of muscles studied.

## Results

### CIM skeletal muscle expresses the Na_V_ 1.5 α subunit and a Na_V_ 1.4 α subunit with altered glycosylation

**Figure 1:**
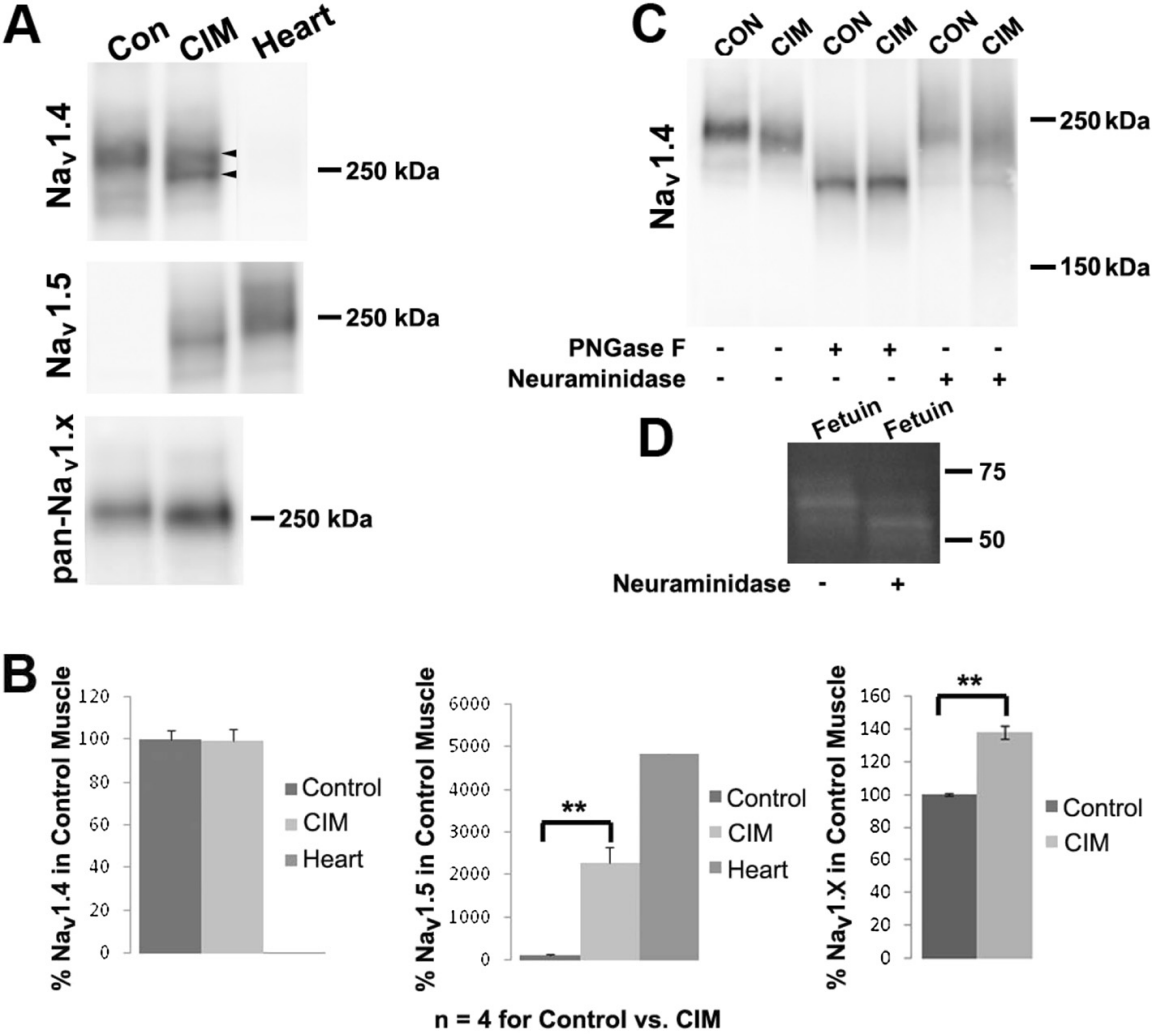
**CIM muscle expresses the Na**_**V**_**1.5 α subunit and a Na**_**V**_**1.4 α subunit altered in its glycosylation.** ( **A**) Western blots were performed on membranes prepared from gastrocnemius muscles of individual control or CIM rats using antibodies specific to the Na_V_ 1.4 and 1.5 sodium channel isoforms or a pan-Na_V_ 1.x antibody. Heart was a positive control for Na_V_ 1.5. The mobility of the Na_V_ 1.4 changed in CIM (see the upper arrow to normal channel versus lower arrowhead pointing to altered). It should be noted that 5% gels were used for the blots with the Na_V_ 1.4 and 1.5 antibodies, while a 4% to 20% gel was used for the Na_V_ 1.x antibody, thus reducing the apparent size difference between control and CIM for that antibody. (**B**) Quantification of blots with different sodium channel antibodies. The average expression of protein under control conditions was set as 100%, and expression in individual control and CIM was calculated relative to this average ( *n* = 4). Error bars shown are SEM, and asterisks indicate *P* < 0.01. Since the total amount of Na_V_ 1.4 does not change in CIM, the increase observed with the pan-Na_V_ 1.x is attributed to the up-regulation of the Na_V_ 1.5. (**C**) Membranes from control and CIM muscles were treated with either PNGase F (to remove all N-linked glycosylation) or Neuraminidase (to remove only terminal sialic acid) and visualized by western blotting with a Na_V_ 1.4-specific Ab on 5% gels. Treatment with PNGase F eliminated the migration difference between control and CIM channel, while Neuraminidase did not. (**D**) Neuraminidase treatment of fetuin, a control protein that is highly sialyated, shifts the molecular weight, indicating that the enzyme was functional.

We utilize a rat model of critical illness myopathy (CIM) in which corticosteroid treatment is combined with denervation to mimic treatment of critically-ill patients with corticosteroids and neuromuscular blocking agents [5,6]. Previous analysis of mRNA indicated that CIM skeletal muscle expresses the Na_V_ 1.4 and Na_V_ 1.5 isoforms of sodium channel [15]. To confirm this at the level of the protein, we analyzed western blots of skeletal muscle membrane fractions with antibodies specific to the two channel isoforms (Na_V_ 1.4 and Na_V_ 1.5) and also with a pan-sodium channel antibody (pan-Na_V_ 1.x) to compare the amount of both of these isoforms simultaneously (Figure 1, A and B). Membranes from heart were used both as a negative control for the Na_V_ 1.4-specific antibody and as a positive control for the Na_V_ 1.5 antibody. The change in expression observed with each of these antibodies (Figure 1B) suggests that the increased expression with the pan-Na_V_ 1.x antibody is due to up-regulation of the Na_V_ 1.5 and suggests that the Na_V_ 1.5 represents approximately 28% of the total sodium channel protein in CIM.

While the amount of Na_V_ 1.4 α subunit remained constant, its migration was altered. One potential explanation for altered migration of the Na_V_ 1.4 α subunit is an alteration in the level of glycosylation. The Na_V_ 1.4 α subunit is known to be highly glycosylated [16,17]. Membranes from control or CIM tissue were treated with one of two enzymes, PNGase F, which removes N-linked glycosylation at the ASN-linkage [18] or a recombinant neuraminidase, which removes terminal sialic acid moieties [19]. The samples were then probed in a western blot with the Na_V_ 1.4-specific antibody. As shown, removal of the entire carbohydrate sugar tree at the N-linkage completely abolished migration differences between the control and CIM samples, while treatment with neuraminidase did not (Figure 1C). As a positive control for neuraminidase function, we treated fetuin (a protein with a large number of sialic acid moieties), under the same conditions and found a shift in migration (Figure 1D). Taken together, these data indicate that a focused removal of terminal sialic acid moieties is insufficient to account for the change in migration of Na_V_ 1.4.

### Identification of a phosphorylation site in the Na_V_ 1.4 α subunit

In the Na_V_ 1.2 brain sodium channel, a complex interplay of phosphorylation of sites within the I-II loop and the III-IV loop carried out by protein kinase A and C reduce peak sodium currents [20]. Skeletal muscle sodium channels are not as highly phosphorylated as their brain and neuronal counterparts [21], but phosphorylation could affect gating of sodium channels in CIM. Na_V_ 1.4 is phosphorylated by protein kinase A *in vitro* at a single site [21]. In the Na_V_ 1.5 channel, phosphorylation of S1505 in the III-IV loop by protein kinase C both reduces peak current and shifts inactivation gating in the hyperpolarizing direction [22].

**Figure 2:**
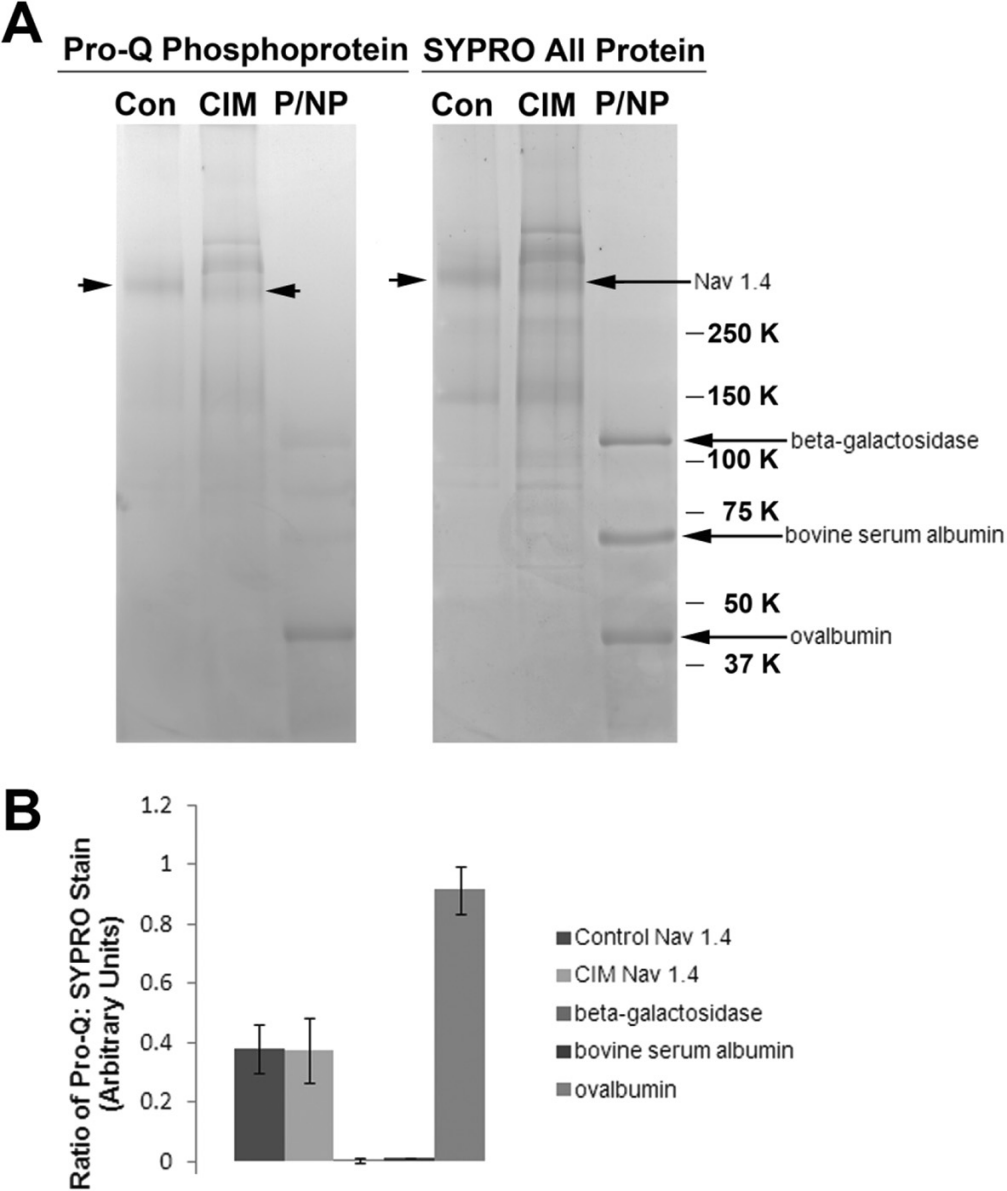
**The Na**_**V**_**1.4 is phosphorylated at similar levels in control and CIM muscle.** ( **A**) To assess the degree of phosphorylation in CIM and control muscle, classically-purified sodium channel was stained with a dye that binds only phosphoproteins (Pro Q Diamond phosphoprotein dye) and the ratio of signal with this stain was compared to that of a dye that binds all proteins (SYPRO Ruby Red protein stain). As controls, a set of proteins that are non-phosphorylated (β-galactosidase, bovine serum albumin) or a known phosphoprotein (ovalbumin) were analyzed on the same gel (P/NP markers). Of these controls, only the ovalbumin stained on both gels, indicating that staining conditions were optimal for each dye. Control and CIM sodium channel, indicated by arrows, had similar phosphostaining. ( **B**) Quantification of the ratio of Pro Q: SYPRO protein stains for each of the experimental and control proteins. The control and CIM proteins had very similar degrees of phosphorylation, while only the ovalbumin standard was phosphorylated.

**Figure 3:**
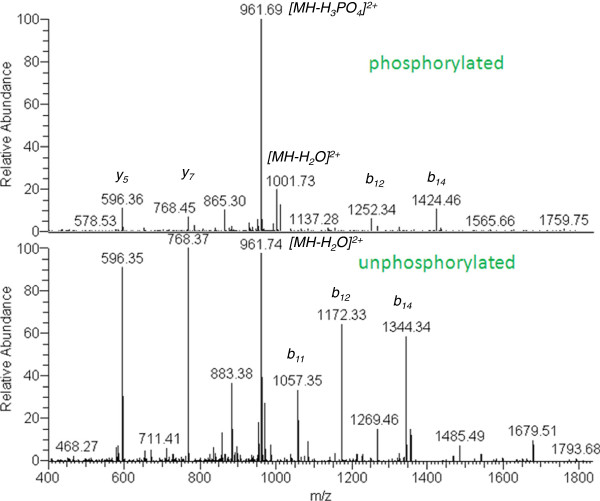
**Identification of Ser**^**487**^**as a phosphorylation site in Na**_**V**_**1.4 channel.** Using previously reported methods [11], a purified fraction of sodium channels was prepared from control and CIM muscles and the excised band corresponding to the Na_V_ 1.4 α subunit was excised, trypsinized, and analyzed by tandem mass spectrometry. This analysis indicated that the serine at 487 was partially phosphorylated, as we detected both the phosphorylated and unmodified forms in the samples. The MS/MS spectra of both forms were shown. The main product ions (*b* and *y* ions) were indicated. It is clear that a number of *b* ions ( *b*_12_ and *b*_14_) from the two peptides differ in a mass of 80 Da, the mass shift due to phosphorylation. In addition, neutral loss of phosphorus group was also observed for the modified form. The spectra shown are from a control sample. Although both control and CIM spectra were partially phosphorylated at the Ser^487^ site, the exact percentage was difficult to determine due to the small amount of sodium channel analyzed.

As an assessment of the degree of overall phosphorylation, classically-purified control and CIM sodium channels [11] were comparatively stained with Pro-Q Diamond Phosphoprotein Stain (which stains only phosphoproteins) and SYPRO Ruby Protein Stain (which stains all proteins) (Figure 2A). Quantitative comparison of the fluorescent signal intensities of the two dyes was made, and the ratio of Pro-Q to SYPRO was found to be constant in control versus CIM channel (Figure 2B). To determine the site at which the phosphorylation occurred, the sodium channel bands were excised from control and CIM samples, trypsinized, and analyzed by tandem mass spectrometry (Figure 3, control sample shown). The serine at position 487 was partially phosphorylated and lies within the general region previously found to contain an *in vitro* cAMP-phosphorylation site [21]. However, its surrounding sequence [QALES*GEE] does not correspond to the conserved consensus sequence of [RK] 2x [ST] for either cAMP or cGMP-dependent protein kinase. The lack of a quantitative difference between the control and CIM channels, based on the ratio of Pro Q: SYPRO (Figure 2B), suggests that gross changes in levels of phosphorylation do not underlie the shift in voltage dependence of inactivation.

### No difference in FGF sub-type associated with CIM sodium channel

**Figure 4:**
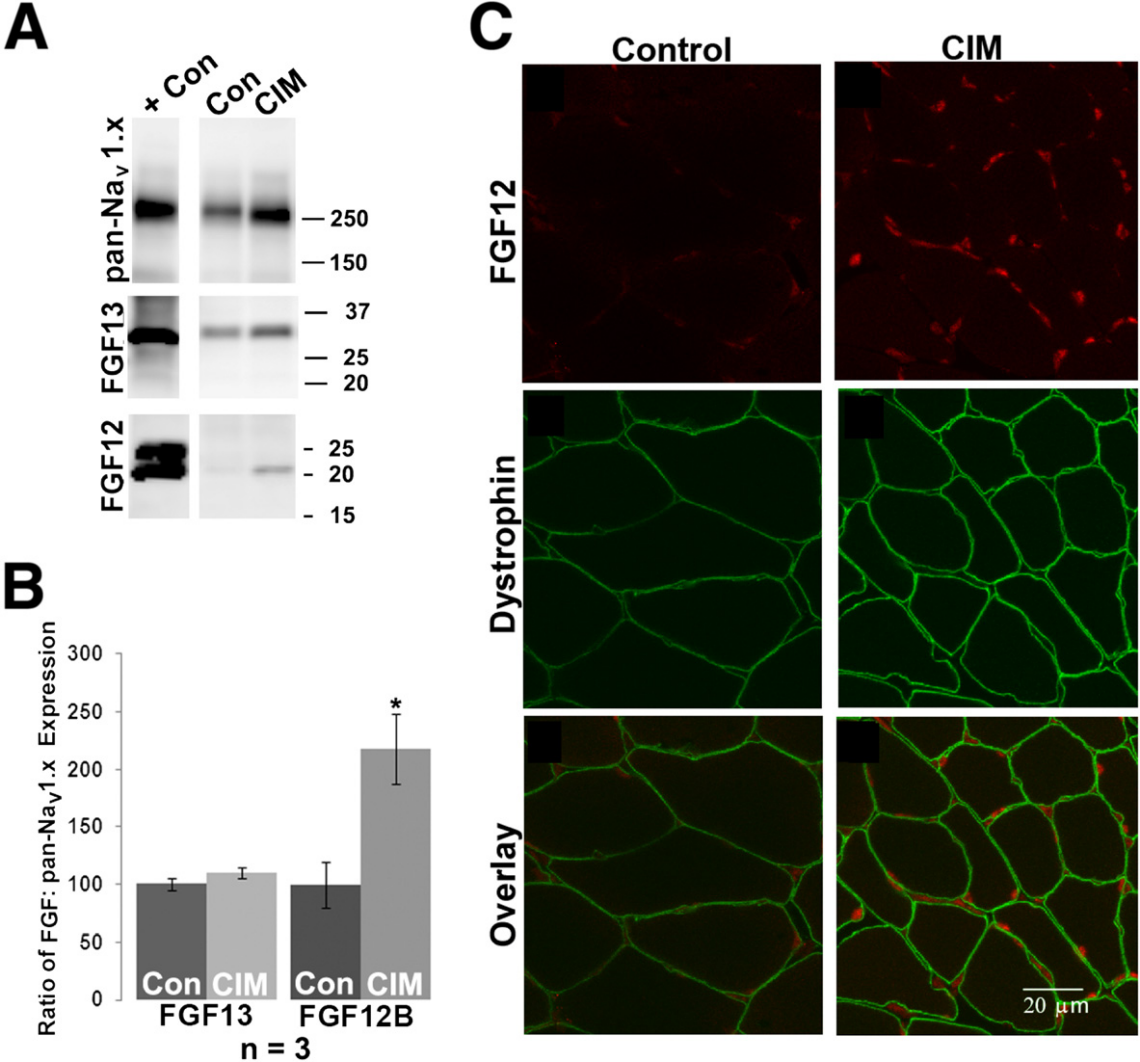
**FGFs appear unlikely to contribute to altered sodium channel gating in CIM.** ( **A**) FGF12 and 13 levels in muscle membrane samples from control and CIM muscle. As positive controls, tissues or expression vectors known to produce the protein of interest were used: for the pan-Na_V_ 1.x Ab, skeletal muscle; for FGF13, a lysate of 293 cells transfected with an FGF13 expression vector; for FGF12, brain membranes. (**B**) FGF13 was expressed in both control and CIM muscle, such that the relative ratio of FGF13: sodium channel did not change in CIM. In contrast, FGF12B was expressed at low levels in control muscle, but increased in CIM muscle, such that the ratio of FGF12B: sodium channel significantly increased. Asterisk indicates *P* < 0.05. ( **C**) Cross-section of control and CIM medial gastrocnemius muscle stained for FGF12, dystrophin, and overlay reveals that most FGF12 staining is in myonuclei and nuclei of cells in the interstitial space.

Fibroblast growth factors (FGFs) have emerged as a class of proteins that modulate sodium channel gating [23-25]. Different FGF isoforms/splice variants shift the inactivation gating curves of sodium channels to varying degrees, such that a switch in expression of one sub-type over another could alter the voltage dependence of sodium channel inactivation in CIM [23-25]. Thus, relative expression of FGFs may be a mechanism through which cells regulate excitability. Membranes from control and CIM gastrocnemius muscles were analyzed for expression of FGF proteins (Figure 4). While we found that neither control or CIM skeletal muscle expressed FGF 11 and 14 (data not shown), both control and CIM membranes expressed the same ratio of FGF13 relative to the amount of total sodium channel protein detected with pan-Na_V_ 1.x antibody (Figure 4, A and B). In CIM membranes, there was increased expression of FGF12B relative to the pan-Na_V_ 1.x antibody (Figure 4, A and B). However, further analysis of FGF12 localization by staining in muscle cross-sections indicated the protein was expressed in nuclei, not the surface membrane, and thus was unlikely to interact with sodium channels to alter the voltage dependence of inactivation (Figure 4C). Thus none of the FGFs appear to be candidates that could account for the hyperpolarized shift in the voltage dependence of inactivation in CIM.

### The sodium channel is part of a protein complex that is altered in CIM

**Figure 5:**
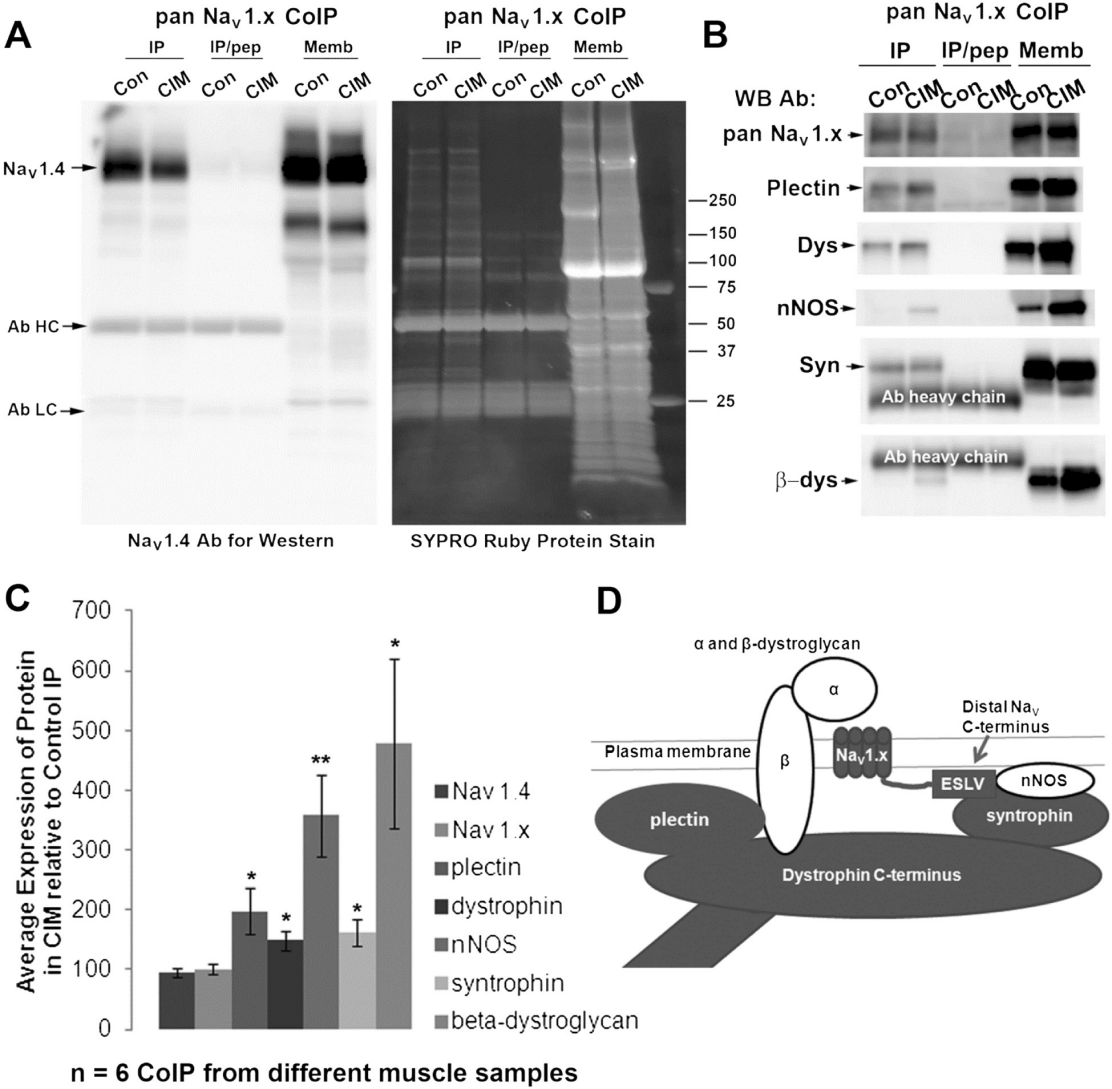
**Na**_**V**_**1.4 co-precipitates with members of the dystrophin associated protein complex (DAPC).** Using an antibody generated against a peptide corresponding to the highly conserved III-IV linker region (pan Na_V_ 1.x), sodium channels from NP40-solubilized control and CIM skeletal muscle membranes were immunoprecipitated. The immunoprecipitated (IP) control (Con) or CIM materials were resolved on SDS-PAGE gels and probed in western blots with the indicated antibodies. As a negative control, the IP was performed in the presence of blocking peptide (IP/pep), the same sequence used as the antigen; and as a positive control, starting membranes were used (Memb). (**A**) A full-panel western with the Na_V_ 1.4-specific monoclonal antibody LD3 is shown on the left in comparison to a protein gel stained with SYPRO Ruby protein stain on the right. The denatured antibody heavy chain (Ab HC) and light chain (Ab LC) are present in the immunoprecipitated samples, as best seen in the SYPRO stained gel. A large number of proteins co-precipitate with the sodium channel (Na_V_ 1.x), and many of these are shown to be specific since they are absent in the peptide control. (**B**) Using antibodies against plectin, dystrophin (dys), neuronal nitric oxide synthase (nNOS), syntrophin (syn), and β-dystroglycan (β-dys) confirms that many components of the DAPC are present in the control and CIM IPs. ( **C**) Quantification of the data from panel B. For each antibody in each CoIP, the signal for the control was set as 100% and the signal for the CIM was determined relative to this. The average of the signals for the CIM: control for each antibody is shown, and error bars are SEM ( *n* =6). For some antibodies, notably the sodium channel antibodies, approximately equal signals were seen in control and CIM CoIPs. For other antibodies, notably dystrophin and syntrophin, there were slightly elevated amounts of these proteins present in the CIM. Finally, for the nNOS and β-dystroglycan, there was considerably more protein in the CIM CoIPs. These results are summarized in cartoon form in ( **D**) which shows ‘tightly’ associated proteins in grey, and ‘loosely’ associated proteins in white. The protein associations shown in the cartoon are based on work carried out by a number of investigators, which previously demonstrated that Na_V_ 1.x channels associate with proteins of the DAPC through their consensus S/TXV-COOH C-termini [26,27]. The dynamic regulation of the signaling protein nNOS in CIM suggests that it may play a role in the disease process, including affecting the inactivation gating of the adjacent sodium channels.

Work carried out by a number of investigators has shown that sodium channels are part of the dystrophin associated protein complex (DAPC) [26,27]. To determine if the constituent members of this sodium channel complex are dynamically regulated in CIM, we immunoprecipitated the complex from control versus CIM muscle membranes solubilized in the non-ionic detergent NP40. We used the pan-Na_V_ 1.x antibody, directed towards the highly conserved III-IV linker, to bring down both the Na_V_ 1.4 and 1.5 isoforms. The recovery of sodium channel in the immunoprecipitates (IPs) was specific since a blocking peptide prevented IP of the sodium channel in both control and CIM samples (Figure 5). The starting membranes were used as a positive control. Co-immunoprecipitation (CoIP) of syntrophin and dystrophin, the proteins most closely linked to sodium channel in the complex, was relatively high in both control and CIM samples (Figure 5B and D). Plectin, which is associated with the sodium channel both through its interaction with β-dystroglycan and dystrophin [28], is present at lower levels in control than CIM samples. Other proteins (β-dystroglycan and nNOS) were present primarily in the CIM samples (Figure 5B and D). A number of CoIPs were carried out to assess the degree to which proteins were present and absent in the control versus CIM complexes, such that statistical significance was reached (Figure 5C). The cartoon summarizes the dynamic nature of the complex (Figure 5D), in that β-dystroglycan and nNOS, shown in white, are primarily associated with CIM sodium channels.

**Figure 6:**
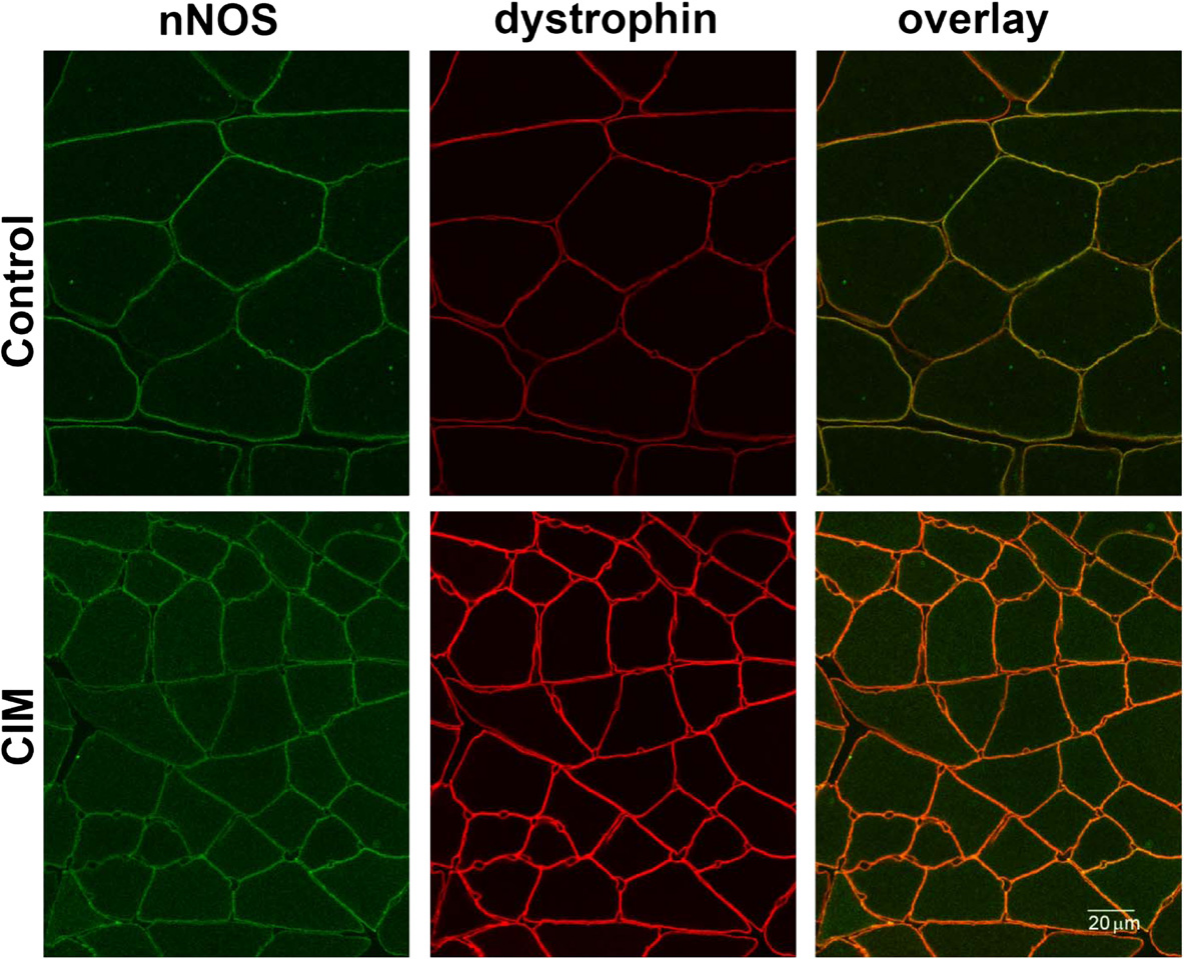
**nNOS is expressed in the sarcolemma of control and CIM muscle.** Cross-sections of medial gastrocnemius muscle were stained with the antibodies to nNOS as well as dystrophin to mark muscle surface membrane. In both control and CIM, nNOS is expressed in the surface membrane. The myofiber cross-sections are much smaller in CIM muscle, due to the previously reported muscle atrophy in the disease [10].

In other models of muscle atrophy, nNOS was reported to move from the surface membrane to an intracellular pool [29]. To confirm that nNOS is co-localized with sodium channel in the surface membrane in the CIM muscle, cross-sections of CIM muscle were stained for nNOS using dystrophin as a marker for the surface membrane (Figure 6). Both control and CIM surface membrane were positive for nNOS (Figure 6). Taken together, the biochemical and immunostaining data indicate that nNOS is a dynamically regulated member of the sodium channel-DAPC complex.

### nNOS plays a role in loss of muscle excitability following denervation

**Figure 7:**
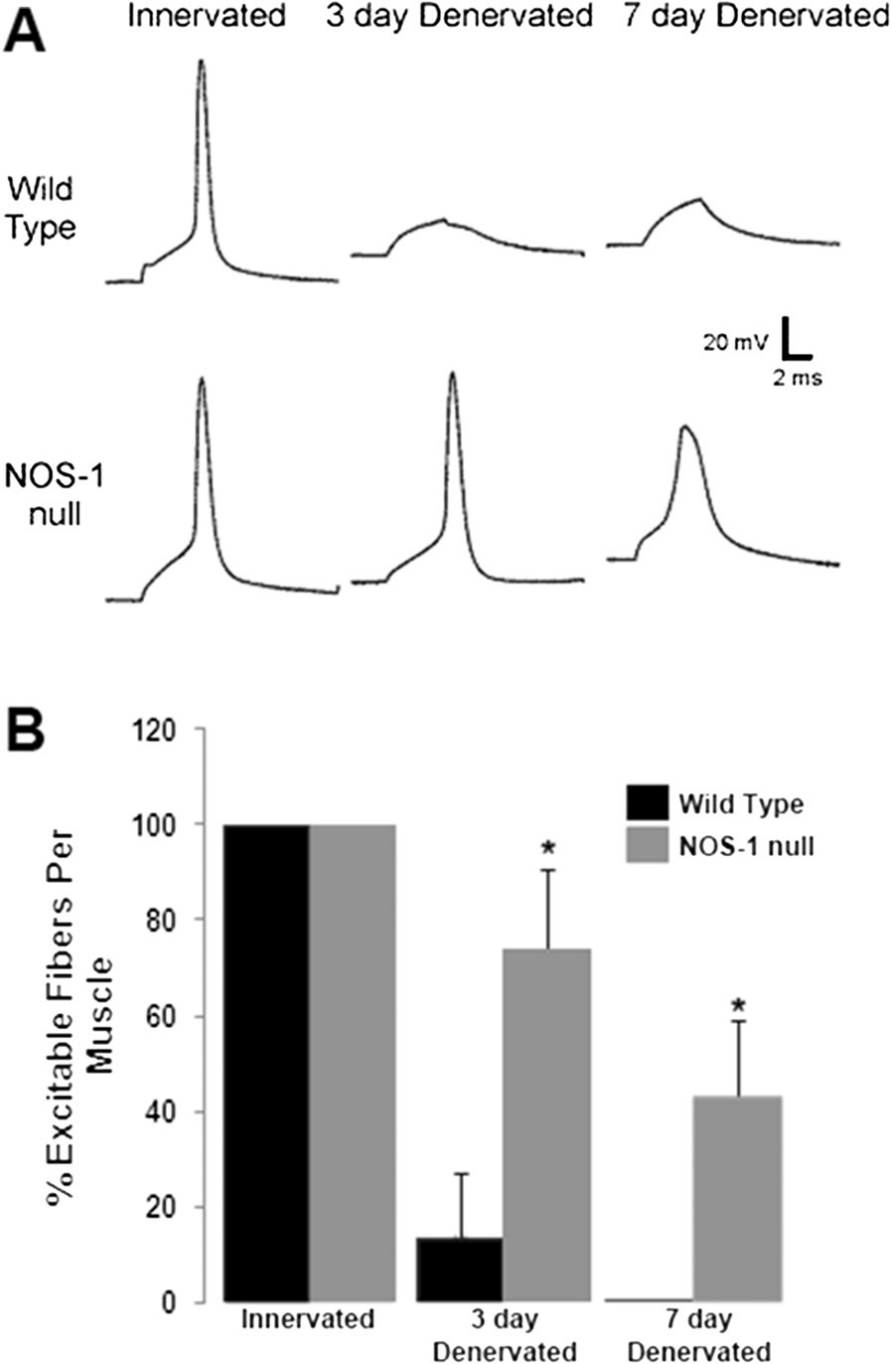
**Muscle lacking nNOS is more excitable following denervation.** ( **A**) Action potential traces from innervated and denervated wild-type and nNOS (NOS-1) null fibers. In innervated wild-type and nNOS null fibers, all or none action potentials are present. In the control 3 and 7 day denervated traces shown, no action potential is present and the only response is passive depolarization of the membrane potential in response to current injection. In the 3-day denervated nNOS null fiber shown a nearly normal action potential is present. In the 7-day denervated nNOS null fiber shown an action potential is present, but it is smaller and wider than action potentials from innervated muscle. ( **B**) Plot of the percent of excitable fibers in innervated and denervated wild-type and nNOS null fibers. In both wild-type and nNOS null innervated muscles 100% of fibers were excitable in all muscles studied ( *n* = 4 for wild-type, *n* = 3 for nNOS null). Three days following denervation only 13% of fibers were excitable in wild type muscles ( *n* = 4). In nNOS null muscles 73% of fibers were excitable 3 days following denervation ( *P* < 0.05 *vs.* wild-type, *n* = 4). Seven days following denervation 0% of wild-type fibers were excitable ( *n* = 4) while 43% of fibers from nNOS null muscles were excitable ( *P* < 0.05 *vs.* wild-type, *n* = 4).

In the rat model of CIM, treatment of denervated muscle with corticosteroids *in vivo* results in inexcitability of muscle fibers [7,8,30]. The biochemical data presented above are consistent with the possibility that increased association of nNOS with sodium channels contributes to loss of excitability. To determine the role of nNOS in regulation of muscle excitability following denervation, we recorded from muscle fibers in nNOS-null and control mice. In innervated mouse muscle, 100% of fibers were excitable (*n* = 4 muscles). We found that denervation of mouse muscle in the absence of treatment with corticosteroids was sufficient to induce inexcitability. In control muscle denervated for 3 days, few muscle fibers were excitable ( *n* = 4 muscles, Figure 7). In contrast, in nNOS-null mice the majority of fibers remained excitable 3 days after denervation (*n* = 4 muscles, *P* < 0.05 *vs.* control). To determine whether absence of nNOS lessened development of inexcitability at a longer time point, we measured excitability in a second set of mice in which muscle was denervated for 7 days. In control muscle, no fibers remained excitable 7 days following denervation ( *n* = 4 muscles); whereas in nNOS-null fibers, excitability was preserved in a fraction of fibers ( *n* = 4 muscles, *P* < 0.05 *vs.* control). These data are consistent with the possibility that increased association of nNOS with sodium channels following denervation contributes to development of muscle inexcitability.

## Discussion

We compared biochemical properties of control and CIM sodium channels to find candidates that might account for the hyperpolarized shift in inactivation gating seen in the acute phase of CIM. We identified several biochemical changes in sodium channel in CIM, but the most promising candidates appeared to be alterations in sodium channel-associated proteins in CIM. In particular, nNOS is a promising candidate that was more associated with sodium channels from CIM muscle. In mice lacking nNOS, the normal reduction in excitability following denervation was greatly reduced. These data are consistent with the possibility that increased association of nNOS with sodium channels is involved in triggering loss of muscle excitability in CIM.

We identified an increase in Na_V_ 1.5 in CIM muscle such that it is approximately 28% of the entire channel population. This is similar to our earlier estimate of 21% obtained by measuring current densities [9]. In our previous study, both the TTX-insensitive (Na_V_ 1.5) and TTX-sensitive (Na_V_ 1.4) channels demonstrated similar hyperpolarizing shifts in inactivation gating in CIM [9], so increased expression of Na_V_ 1.5 *per se* cannot be responsible for the shift.

A second change identified in membranes from CIM muscle was reduced glycosylation of the Na_V_ 1.4 sodium channel. Removal of the entire carbohydrate ‘tree’ at the asparagine-linkage eliminated the molecular weight difference between the control and CIM channel. However, selective removal of sialic acid moieties with neuraminidase did not eliminate the size difference. Given that the Na_V_ 1.4 channel is known to have multiple carbohydrate trees [16,17], the simplest explanation for these observations is that some but not most of the carbohydrate trees are removed in CIM, removing some but not most of the sialic acids. Previous work shows that removal of sialic acid from sodium channels shifts inactivation gating in a depolarizing direction [16,17,31]. This is opposite of the hyperpolarizing shift we observed in CIM [9]. Thus, removal of some carbohydrate trees may be a compensatory mechanism that moves the voltage dependence of inactivation towards more depolarized potentials.

One change we found in CIM muscle that could underlie the hyperpolarized shift in the voltage dependence of inactivation was an alteration in the composition of the dystrophin protein associated complex (DAPC) as summarized in Figure 5D. This figure is based not only on work in this paper, but also on work carried out by other investigators that identified the components of the DAPC (reviewed in [32]). In our hands, the DAPC appeared to dissociate more easily in control samples such that all components (except sodium channels) were present at higher levels in the CIM samples. This was especially true for β-dystroglycan and nNOS, which are present at much higher levels in CIM CoIPs. These observations suggested to us that the sodium channel-DAPC complex is bound more tightly in CIM, perhaps indicating that the sodium channel and cytoskeleton are in a different and more strongly ‘locked’ conformation in the disease. Alternatively, the constituent members of the DAPC may be dynamically regulated. In either case, the presence of the important signaling protein nNOS in the DAPC of CIM muscle suggests that NO signaling through this protein could contribute to the altered inactivation gating in CIM.

The protein components that we identified in the DAPC are consistent with those identified by other investigators [26,27]. In skeletal muscle, the consensus C-termini (S/TXV-COOH) of Na_V_ 1.4 and 1.5 sodium channels bind the PDZ domain of syntrophin at a site overlapping and/or closely adjacent to the binding site for nNOS [26]. Through syntrophin, both sodium channels and nNOS bind the C-terminus of dystrophin [26]. In cardiac muscle, the dynamic nature of this complex was shown by comparative analysis of control *vs.* syntrophin point mutation that causes Long QT syndrome. The syntrophin point mutation altered the complex constituents, such that the plasma membrane Ca^2+^ ATPase no longer bound syntrophin. This released inhibition of nNOS, allowed S-nitrosylation of the Na_V_ 1.5 sodium channel, and altered gating [27].

Dystrophin is part of the muscle cytoskeletal system. In mdx mice, which lack dystrophin, sodium channel inactivation gating is shifted 10 mV more positively than that of control mice [33]. This observation suggests that loss of cytoskeletal components shifts inactivation gating in a depolarizing direction, a finding consistent with our hypothesis that sodium channel inactivation gating is hyperpolarized in CIM because it is more tightly associated with cytoskeletal components. However, acute disruption of cytoskeleton by pressure during formation of seals during patch clamp measurements has been found to trigger hyperpolarized shifts in the voltage dependent of Na_V_ 1.4 and Na_V_ 1.5 activation and fast inactivation [34-37]. Thus, while it is clear that changes in cytoskeleton can have profound effects on the voltage dependence of sodium channel gating, we currently do not know which changes in cytoskeleton will translate into changes in sodium channel gating.

Our finding that nNOS is present at higher levels in the sodium channel-DAPC complex in CIM raises the possibility that increased signaling through NO-dependent pathways contributes to loss of muscle excitability in CIM (see however, [38]). There are several cell signaling pathways that are regulated by NO, including protein phosphorylation through cGMP-protein kinase [39] and direct nitrosylation of cysteine or other amino acid side chains, as discussed above for the cardiac Na_V_ 1.5 [27]. We measured phosphorylation changes in CIM and found no overall difference (Figure 2).

To determine whether increased association of nNOS with sodium channels could be involved in inducing inexcitability of muscle, we measured excitability following denervation in control and nNOS-null mice. In rats, denervation alone induces inexcitability in only a minority of fibers, so addition of corticosteroids is necessary to induce inexcitability [8,30]. In control mice, denervation alone was sufficient to induce inexcitability so it was not necessary to co-administer corticosteroids. In nNOS-null mice, a greater percentage of muscle fibers remained excitable following denervation. There are multiple mechanisms that could account for maintenance of excitability following denervation in the absence of nNOS. Further study will be necessary to determine if the contribution of nNOS to inexcitability is mediated by its association with sodium channels as part of the DAPC.

## Conclusion

We surveyed sodium channels and their associated proteins in control versus CIM muscle using a variety of biochemical techniques to identify candidates that could underlie the hyperpolarized shift in inactivation gating/loss of electrical excitability that is characteristic of CIM. While we identified a number of changes in CIM, including increased expression of the Na_V_ 1.5 sodium channel and partial de-glycosylation of the Na_V_ 1.4 sodium channel, it is the change in association of the sodium channels with members of the DAPC that seems most promising as a potential explanation for the shift in inactivation.

## Abbreviations

CIM, Critical illness myopathy; DAPC, Dystrophin associated protein complex; DTT, Dithiothreitol; EDTA, Ethylenediaminetetraacetic acid; EGTA, Ethylene glycol-bis(2-aminoethylether)-N,N,N’,N’-tetraacetic acid; FGF, Fibroblast growth factor; FHF, Fibroblast growth factor homologous factors; IP, Immunoprecipitation; nNOS, Neuronal nitric oxide synthase; NO, Nitric oxide; NP40, Nonidet P-40; PBS, Phosphate-buffered saline; PMSF, Phenylmethylsulfonyl fluoride; PNGase F, Peptide: N-Glycosidase F; SDS-PAGE, Sodium dodecyl sulfate polyacrylamide gel electrophoresis.

## Competing interests

None of the authors have competing interests.

## Authors’ contributions

SDK carried out all biochemical analyses on channel samples, prepared figures relating to that data, and co-wrote the manuscript. KRN carried out all surgical and drug treatments of animals as well as electrophysiologic recordings. QW carried out immunostaining, analyzed data, and prepared related figures. JP carried out and did all analysis and interpretation on tandem mass spectrometry. MMR supervised all experiments, co-wrote the manuscript, and provided final interpretation of all data. All authors read and approved the final manuscript.
